# A joint complex network and machine learning approach for the identification of discriminative gene communities in autistic brain

**DOI:** 10.1371/journal.pone.0334181

**Published:** 2025-11-05

**Authors:** Antonio Lacalamita, Ester Pantaleo, Alfonso Monaco, Loredana Bellantuono, Alessandro Fania, Marianna La Rocca, Tommaso Maggipinto, Sabina Tangaro, Nicola Amoroso, Roberto Bellotti

**Affiliations:** 1 Dipartimento Interateneo di Fisica M. Merlin, Università degli Studi di Bari Aldo Moro, Bari, Italy; 2 Istituto Nazionale di Fisica Nucleare (INFN), Sezione di Bari, Bari, Italy; 3 Dipartimento di Biomedicina Traslazionale e Neuroscienze (DiBraiN), Università degli Studi di Bari Aldo Moro, Bari, Italy; 4 Dipartimento di Scienze del Suolo, della Pianta e degli Alimenti, Università degli Studi di Bari Aldo Moro, Bari, Italy; 5 Dipartimento di Farmacia—Scienze del Farmaco, Università degli Studi di Bari Aldo Moro, Bari, Italy; Fondazione Policlinico Universitario Gemelli IRCCS, ITALY

## Abstract

Autism is a genetically and clinically very heterogeneous group of disorders. Gene co-expression network analysis can help unravel its complex genetic architecture through the identification of communities of genes that are dysregulated. Using a publicly available brain microarray dataset (experiment GSE28475), we performed a gene co-expression analysis based on Leiden community detection to identify stable communities of genes and used them within a robust machine learning framework with feature selection. We reached an accuracy as high as (98±1)% in discriminating between autism and control subjects and validated our results on an independent microarray experiment obtaining an accuracy of (88±3)%. Furthermore, we found two communities of 43 and 44 genes that were enriched for genetically associated variants and reached an accuracy of (78±5)% and (75±4)% on the independent set, respectively.

An eXplainable Artificial Intelligence analysis on these two causal communities confirmed the pivotal role of autism specific variants thus independently validating our analysis. Further analysis on the restricted number of genes in the identified communities may reveal essential mechanisms responsible for autism spectrum disorder.

## Introduction

Autism spectrum disorder (ASD) is a neurodevelopmental disorder defined by critical problems in social interaction, impaired communication (both verbal and non-verbal), and the presence of restricted and repetitive behavior or interests [1]. Approximately 1% of children are diagnosed with autism spectrum disorder around the world, with males consistently outnumbering females [2].

There is strong evidence of a mainly genetic contribution to autism and of negligible shared environmental effects [3]. However, ASD is genetically and clinically very heterogeneous and this poses a huge and complex challenge. In fact, ASD genetic risk is largely yet to be determined, despite numerous studies of common genetic variations, spontaneous mutations and inheritance patterns [4]. Given this complexity, there is growing interest in the identification of common biological pathways underlying autism.

Transcriptomics can help unravel this complexity and advance our understanding of autism by integrating genetic information with information on genome function; in particular, gene co-expression network analysis can identify communities of genes that are dysregulated in autism [5]. While trascriptomics of brain tissues collected from living samples is not always practical, autism is a developmental disorder of the brain and there are advantages to post-mortem brain studies due to the nature of the pathology [6]. Gene expression studies can be useful to investigate which genes are dysregulated in ASD [7]. Voineagu et al. performed differential expression analysis from postmortem brain tissue of three regions previously implicated in autism and from control samples using microarray assays [5]. They found that gene expression differences were more pronounced in one region, the cerebral cortex, and validated they results on an independent dataset and with RT-PCR. With additional gene co-expression analysis and with testing for enrichment of autism genetic association signals they identified a possibly causal neuronal module of genes. Ginsberg et al. analysed brain tissues of children with idiopathic autism and controls using gene expression and DNA methilation microarrays [8]. They performed differential expression and GO enrichment analysis identifying a set of down-regulated genes involved in mitochondrial oxidative phosphorylation and in protein translation; additionally they confirmed differential expression of genes of synapse formation/function and of cortical development. Their methylation analysis instead could not identify any promoter/gene expression relationship thus suggesting that other mechanisms are responsible for the observed transcriptional changes. By performing gene co-expression analysis they found that three gene modules were significantly associated with the social and/or behavior interaction domain of the ADI-R and enriched for purinergic signaling/immune response, inflammation/immune response, and myelin/myelination signals. A study conducted by Sciara et al. highlighted that the gene expression levels of anti- and pro-inflammatory signaling molecules, measured in both gray and white matter tissue, presented significant differences among samples of ASD and healthy donors [9].

In the field of autism spectrum disorder, community detection has proven to be an effective methodology that can partition input data into multiple sub-structures thus revealing latent functions. The most studied application in this field is the analysis of MRI data, see for instance [10]; other applications include the analysis of demographic, psychological, and lifestyle factors [11], protein interaction networks [12], RNA sequencing data [13] as well as microarray data [14]. Co-expression networks and methods such as WGCNA revealed gene modules enriched for immune and synaptic functions associated with ASD [15]. Voineagu et al. [16] performed a weighted gene coexpressionn network analysis (WGCNA) on individuals with ASD and controls highlighting convergent molecular abnormalities in ASD. Furthermore, Parikshak et al. [17] using WGCNA found that ASD risk genes are enriched in specific modules related to synaptic function and neuronal signaling. However, few studies use advanced community detection algorithms, such as the Leiden algorithm, to analyze transcriptomic complexity in ASD.

In a complex disorder like autism, machine learning techniques can play a crucial role in elucidating the link between gene expression and ASD. These computational methods eliminate the need for pre-existing assumptions about the model, making it possible to reveal intricate relationships. Several studies have used the combination of machine learning and gene expression to shed light on the nature and characteristics of autism [18–23]. Lin et al. applied two machine learning algorithms to identify clusters of ASD patients with homogeneous clinical features using differentially expressed regions of the brain [18]. Gök developed a machine learning model, based on a Bayesian network algorithm and brain developmental gene expression data, to classify with good performance ASD risk genes [19]. Instead Brueggemaan et al. used a machine learning ensemble method fed with brain gene expression and heterogeneous data, to evaluate and rank each gene’s involvement in the etiology of autism [20]. A recent study by Alkhateeb et al. [22] evaluated a range of classical machine learning models—AdaBoost, Random Forest, Support Vector Machines, and Gaussian Naïve Bayes—on transcriptomic data to classify ASD and non-ASD individuals. Their work demonstrated the effectiveness of these models and highlighted that careful preprocessing and model selection can achieve promising diagnostic performance. Tylee et al. implemented a combined approach of co-expression networks and machine learning algorithms applied to blood transcriptome data obtained from seven microarray studies of individuals with and without ASD. Their metodology performed with moderate accuracy and emphasised gender differences in the ASD-related transcriptomic signature [21].

Vidya et al. [24] developed a deep learning model to classify ASD using resting-state functional Magnetic Resonance Imaging data; they incorporated XAI techniques to interpret the predictions of the model, allowing the identification of critical brain regions associated with ASD. This approach not only improved classification accuracy, but also provided insights into the neural underpinnings of ASD, enhancing the clinical relevance and trustworthiness of the model.

We used microarray transcriptome data from post-mortem brain samples [16,25,26] and a community detection method based on the Leiden algorithm [27] to identify gene communities that are predictive of ASD. We constructed the communities with a hierarchical procedure and fed them to a robust machine learning framework with feature selection [28] to discriminate ASD vs control samples. Also we performed an eXplainable Artificial Intelligence (XAI) analysis [29,30] to allocate credit for our model’s output among genes in a gene community.

The paper is structured as follows. In the “Materials and Methods" section we describe the adopted preprocessing procedure and the applied methodology. In the “Results" section, we report our findings. In the “Discussion" section, we summarize and discuss our results and also highlight certain limitations of our study.

## Materials and methods

**Fig 1:**
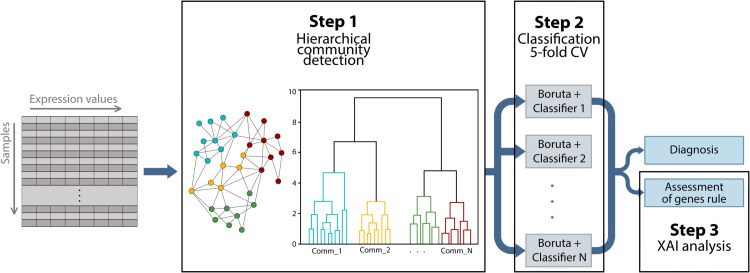
Flowchart of the proposed pipeline. Initially, a gene co-expression network is constructed based on significant Pearson’s correlations between gene expression profiles. In Step 1, hierarchical community detection using the Leiden algorithm is applied to identify stable and biologically relevant communities within the network. These communities serve as the basis for the independent machine learning analysis performed in Step 2, which consists of a 5-fold cross-validation procedure that includes Boruta feature selection and a Random Forest classifier to discriminate between ASD and control subjects. Finally, in Step 3, XAI analysis based on Shapley values is conducted to interpret the classifier results by quantifying the contribution of each gene within the identified communities, enhancing biological interpretability of the predictive model.

Our methodology consists of three main steps illustrated in Fig 1.

In the first step we built the gene co-expression network and implemented a hierarchical and stable community detection procedure using the Leiden algorithm. In the second step we applied a learning pipeline based on a 5-fold Cross Validation (CV) procedure, Boruta feature selection and the Random Forest algorithm to classify ASD vs control subjects using each of the communities identified in the first step. In the last step we implemented the Shapley Additive Explanations (SHAP) method to measure and quantify the contribution of each gene to the classification model. This work applies a previously validated pipeline originally developed for liver cancer analysis [31] to assess the generalizability of combining complex network analysis, machine learning, and XAI by applying it to a different context of gene expression: Autism Spectrum Disorder.

### Data sources

We downloaded two microarray datasets from GEO and used one of them for training and the other as an independent test. Both datasets are expression assays from postmortem brain tissue (prefrontal cortex) samples. The first dataset, GSE28475, consists of 104 samples (81 Frozen tissue and 21 formalin-fixed tissue extracted RNA samples) divided in two classes: 33 ASD and 71 control samples [25,26]. The second dataset, GSE28521, contains 58 samples of 29 ASD brain tissues and 29 control tissues after filtering for high quality data [16]. Diagnostic criteria of autistic disorder was verified for all autistic cases by review of psychological and medical records, including ADI-R [32] and ADOS [33].

We applied to both datasets a preprocessing procedure to minimize batch effects; we used the *ComBat* function from package *sva* version 3.50 [34], where we evaluated the adjustment coefficients (shifts and scalings) only on the control samples and then we fit the model on the ASD samples to remove any batch effects. Then, we *log*2 transformed and quantile normalized the data with function *lumiN* (using method *quantile*) from package *lumi* version 2.54 [35].

Normalization and batch effect correction were performed following the preprocessing pipeline validated in the original study associated with the dataset [36], which demonstrated that this approach effectively reduces technical variation, including batch-associated effects, while preserving biological signal in postmortem brain samples.

For the entire analysis we used R version 4.2.2 with packages *Bioconductor* version 3.16 [37], *igraph* version 1.4.1 [38], *Boruta* version 8.0 [28], *RandomForest* version 4.7-1.1 [39], and *treeshap* version 0.1.1 [40]. Both the raw and the normalized versions of the dataset are available in the following GitHub repository https://github.com/alacalamita95/Plos_One_ASD/tree/main.

### Community detection procedure

We built a complex network of genes where two genes were linked if the Pearson’s correlation between their expression profiles was significant (at a 99% confidence interval) and links were weighted based on the correlation value [41,42]. Communities within this network were identified using a hierarchical strategy based on multiple iterations of the Leiden algorithm [27]. This approach aimed to uncover gene communities relevant to ASD by applying machine learning models independently to each community resulting from the optimal partition.

Starting from a random configuration of nodes and communities, the Leiden algorithm chooses the possible partitioning of the initial network into subgroups that maximize positive-weight connections within communities and negative-weight connections between communities. Given the algorithm’s inherent randomness, we evaluated the *stability* of the partitions by repeatedly running the algorithm under different random initializations. The challenge of extracting meaningful biological insights from communities containing over 100 genes prompted us to repeatedly employ the Leiden algorithm on the entire co-expression network. To enhance biological interpretability, we sought communities of fewer than 100 genes and discarded those with fewer than 4 genes [43,44]. More specifically, we treated initial communities as second-level co-expression sub-networks and independently applied the Leiden algorithm to each of these sub-networks. We recursively subdivided the resulting communities until all sub-communities satisfied the size constraint.

Through iterative application, the Leiden algorithm converges to a partition where all subsets of the obtained communities are locally optimally assigned. At each level, we selected the most stable partition by adjusting two internal parameters of the algorithm: resolution *γ* and randomness *β*, while keeping all other settings at their defaults. For each configuration (*γ*,*β*) of these internal parameters, we assessed the stability of the community detection outcome by running the Leiden algorithm *L* = 100 times. Each run corresponded to a different seed of the pseudo-random number generator and a distinct initial arbitrary assignment of nodes to groups. In the *j*-th run (j=1,…,L), the algorithm produced a partition *p*_*j*_, and the most frequently occurring (majority) partition across all *L* runs was determined through majority voting. This partition was retained, provided it satisfied three criteria: *stability*, *substantiality*, and *non-fragmentation*.

We evaluated stability based on the similarity among partitions $\{p_{j}{\}}_{j=1,\ldots ,L}$ obtained in the *L* runs, corresponding to different random initial conditions. This similarity was assessed using the average normalized mutual information:

$$\langle NMI\rangle =\frac{2}{L(L-1)}\sum_{a=1}^{L-1}\sum_{b=a+1}^{L}NMI(p_{a},p_{b}),$$ (1)

where $NMI(p_{a},p_{b})$ represents the normalized mutual information between a given pair $\left(p_{a},p_{b}\right)$ of partitions, and L(L−1)/2 is the number of distinct pairs. We retained a partition only if ⟨NMI⟩≥0.80.

Furthermore, the Leiden algorithm’s predominant partition should be *substantial*, that is it should not merely consist of a single community that aligns with the entire network or the sub-network it was applied to.

Additionally, we prevented excessive *fragmentation* by checking that the prevailing partition across multiple runs (denoted as *L*) did not encompass communities with a node count less than 5% of the entire network size. When multiple (γ,β) configurations satisfied all criteria, we selected the one with the highest ⟨NMI⟩ for that hierarchical level.

### Feature selection

After applying the gene community detection procedure described in the previous section, we used the Boruta wrapper method to further reduce the number of genes (input features) in each community. This technique can distinguish between relevant and irrelevant information with the goal of improving the accuracy of the learning model. More specifically, Boruta is a supervised feature selection algorithm based on the Random Forest method. It exploits the principles of Random Forest (RF), where random perturbations in the system and randomisation of the training samples mitigate the adverse effects of inherent random fluctuations and correlations in the learning model [28]. It iteratively removes features proven to be less relevant than random probes, ensuring that only statistically significant variables are retained. In details, Boruta works by adding shuffled copies of all features (shadow features) to the dataset, training a Random Forest model, and then using statistical testing (typically a Z-score) to assess whether each original feature has significantly higher importance than the best-performing shadow feature, thereby filtering out irrelevant variables in a robust and conservative manner.

In this work we proposed a robust feature selection framework that applies the Boruta algorithm within a CV procedure. For each community found in the first step of our pipeline and within each CV fold, we applied Boruta. Then we considered the first *N* most frequent genes selected by Boruta, where *N* is the average number of features marked as important for each CV split and repetition. We applied the Boruta algorithm nested in the CV scheme to select pivotal genes and eliminate redundant and confounding features and evaluated the classification performances of these communities. We determined the cardinality of each community by averaging the number of genes selected by Boruta throughout the CV, with genes ranked by their mean importance.

The identification of gene communities and the subsequent feature selection led to a notable reduction in the overall number of features.

### Machine learning framework

The feature selection procedure was run on 80% of the data and the selected features were then used as input for a Random Forest (RF) classifier model on the same 80% of data for training. We then estimated performances on the remaining 20% of the dataset. We repeated this procedure on 5 folds of CV 100 times.

RF is a widely used machine learning algorithm because it is robust and relatively easy to tune, with only two key parameters: (i) the number of trees (M); and (ii) the number of randomly selected features at each split (s) [45]. The RF algorithm is an ensemble of classification and regression trees (CART) generated by a bootstrap procedure. The inherent randomisation during training provides robustness to overfitting and generates trees with low mutual correlation. In our work, we used a standard configuration with *M* = 300 trees and *s* = $\sqrt{S}$, where *S* is the total number of input features.

### Biological validation

For the biological validation of our findings we tested the identified gene communities for over representation for genetically associated variants as determined by the *SFARI Gene* [46] database. This is an evolving database curated by the Simons Foundation Autism Research Initiative (SFARI) and is centered on genes implicated in autism susceptibility [47]. Also we performed KEGG enrichment analysis [48–50] using the R clusterProfiler [51] package; we considered a function significantly enriched when the Bonferroni corrected p-value was lower than 0.05.

### XAI analysis

Explainable Artificial Intelligence (XAI) methods were born with the aim of improving the transparency and interpretability of machine learning results[52–56]. A XAI framework seeks to integrate informativeness and generalisation, where the generalisation refers to the reliability of predictions given previously unseen data, and transparency aims at making model decisions that are easy to understand [57–62].

In this work, we used a local SHAP (SHapley Additive exPlanations) explanatory algorithm to measure and quantify the contribution of each gene to the ASD vs control classification model. SHAP is based on the concept of Shapley values and cooperative game theory [29,30] and works as a local, model-agnostic post-hoc explainer building a local, interpretable linear models to quantifies the contribution of each feature in the Machine Learning model decision. The calculation of a SHAP value for a specific feature relies on assessing the change in the model’s prediction when that feature is included or excluded, averaging across every possible subset of features. For all possible feature subsets *F*, derived from the comprehensive feature set *S* (F⊆S) considering a feature *j*, the SHAP value is given by the difference between the model output with *j* and the model output without it. The SHAP value of the j-*th* feature for the observation *x* is measured through the addition of the *j*-*th* feature to all possible subsets, The SHAP value of the *j*-th feature for observation *x* is calculated by incrementally adding the *j*-th feature to all feasible subsets:

$$SHAP_{j}(x)=\sum_{F\subseteq S-\{j\}}\frac{|F|!(|S|-|F|-1)!}{|S|!}[f_{x}(F\cup j)-f_{x}(F)],$$ (2)

where |F|! is the number of feature permutations in subset *F*; (|S|−|F|−1)! is the number of feature permutations that follow the *j*-*th* feature value; |S|! represents the total number of feature permutations.

## Results

**Fig 2:**
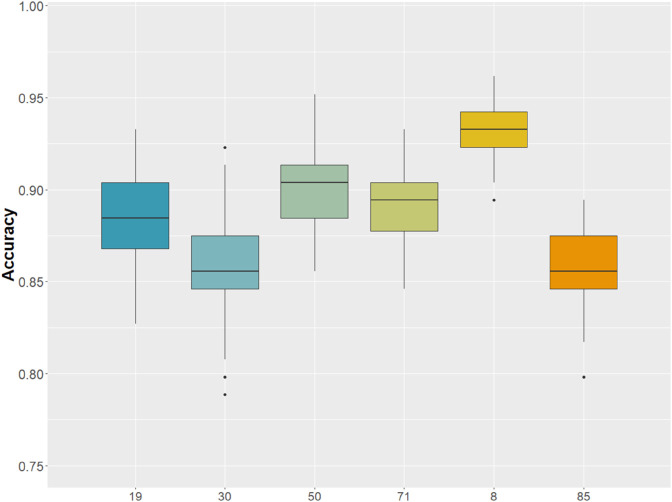
Box-plots of the classification accuracies. The accuracies was obtained from the six gene communities significantly enriched with SFARI database. Each box represents the accuracy distribution obtained by training a Random Forest classifier within a 5-fold cross-validation framework (with Boruta feature selection), repeated over 100 rounds. The numbers below each box represent the number of the corresponding community.

Using a microarray dataset from 104 postmortem brain tissue samples (33 ASD and 71 controls) we built a co-expression network and performed a hierarchical community detection on this network using the Leiden algorithm. We identified 86 stable communities that by design contained a number of genes between 4 and 100 and used each of them to train a random forest model within a 5-fold CV framework with Boruta feature selection. Almost half of the defined communities, namely 41 communities, reached an accuracy greater than 85% in classifying ASD vs controls, with the best performing community reaching an accuracy of 98±1.2%. Furthermore, six of these communities had an over representation of SFARI genes (significant over representation at 1% level, hypergeometric test). In Fig 2, we report the box-plots of the classification accuracy of these six comunities of interest and in Table 1 we report other measures of classification performance computed through a 5-fold CV procedure repeated 100 times, such as area under the curve (AUC), F1 Score and average number of genes selected by Boruta across folds and rounds of the CV. Supplementary S1 Table reports the performance of the complete set of 41 communities, while Appendix A1 provides the list of genes belonging to each of these communities.

**Table 1:** Classification performances. Performance refers to the six gene communities significantly enriched with SFARI database on the training set. The results shown include the mean AUC, the F1 score, and the average number of genes selected by Boruta feature selection in 100 repetitions of 5-fold cross-validation.

Comm	N	AUC (%)	F1 Score (%)	SFARI genes
Comm_8	39	88.8 ± 2.2	93.5 ± 1.5	SOD1, ACTB, ATP1A1, DPYSL3, CSNK2B
Comm_19	34	89.2 ± 2.4	93.7 ± 1.4	KIF5C, LMTK3, CHD8, DPYSL2, TSPAN7
Comm_30	44	88.0 ± 1.9	93.4 ± 1.1	OR2T10, PCDHA6, DEPDC5, DYRK1A
Comm_50	44	92.5 ± 1.4	96.0 ± 0.9	ACHE, GABRB3, PPP1R9B, PBX1, KLF16, WAC
Comm_71	43	92.8 ± 1.5	95.5 ± 1.1	POMT1, RFX4, MYOCD, FRK
Comm_85	41	84.0 ± 2.6	89.7 ± 1.6	SNX5, RPS6KA2, P4HA2, APBB1, PSD3, GRIK4, PHF2

We tested the performance on the independent set of the 41 communities that had accuracy higher than 85% on the training dataset. Of these communities, 38 reached an accuracy greater than 70% on the independent dataset. Comm_78 reached the highest accuracy of 88±3%. Two communities, namely Comm_50 and Comm_71, that also shared 6 and 4 genes with the SFARI database, achieved an accuracy of 78±5% and 75±4%, respectively, see Table 2 which also reports AUC and F1 score. Supplementary Table S2 reports the performance of the set of 41 communities.

**Table 2:** Classification performances of the two found gene communities on the independent test dataset. The table reports mean classification accuracy, the number of overlapping genes, AUC and F1 Score for the independent dataset. Results were obtained by averaging over 100 repetitions of the 5-fold cross-validation procedure, with estimated errors indicated. Detailed results for the complete set of 41 communities are provided in Supplementary S2 Table.

Community	N	Accuracy (%)	AUC (%)	F1 Score (%)
Comm_50	44	78 ± 5	78 ± 5	79 ± 5
Comm_71	43	75 ± 4	75 ± 4	77 ± 5

**Fig 3:**
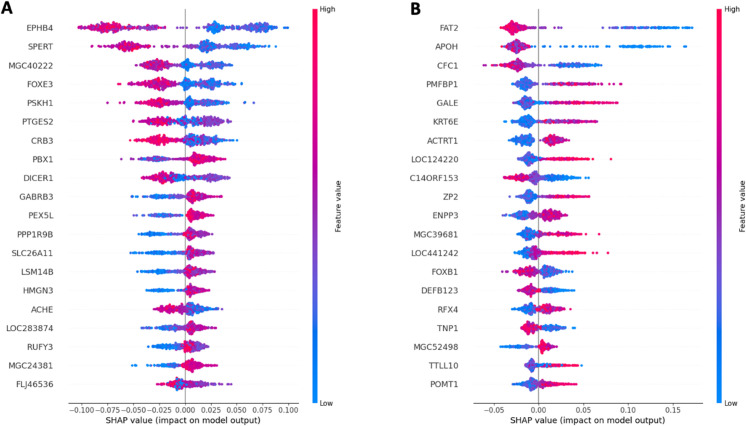
SHAP summary plots of the two found communities on the independent test dataset. The plots illustrate how individual gene expression values influence the classifier’s prediction of ASD. Each row corresponds to a gene, ordered vertically by importance (the top 20 most influential genes are shown). The horizontal axis represents the SHAP values, indicating the magnitude and direction of each gene’s impact on the prediction: positive SHAP values correspond to contributions towards an ASD-positive diagnosis, while negative values contribute to a control diagnosis. Each point represents a sample, colored according to the expression level of the corresponding gene (low expression in blue, high expression in red).

We performed further analyses on Comm_50 and Comm_71 containing autism variants. First we performed KEGG enrichment analysis and found that Comm_50 was enriched for the glycosylphosphatidylinositol-anchor biosynthesis pathway and that Comm_71 was enriched for the fatty acid elongation pathway. Then, we performed SHAP analysis. In Fig 3, we represent the resulting SHAP plots which illustrate the relationship between the classification outcome and individual gene expression for these two communities; rows correspond to genes, and only the firsts 20 most important genes are represented (most important genes at the top). The SHAP summary plot conveys information about features and their effects. In this representation, each dot represents a Shapley value associated with a feature and an instance. On the y-axis are indicated the features, while the x-axis reflects the Shapley value. In addition, the color indicates the value of the feature, which ranges from low to high. To avoid overlaps, the points along the vertical axis are slightly scattered and give an insight into the distribution of Shapley values per characteristic. The characteristics are ordered according to their importance. Therefore a gene expression value with Shapley score greater than zero is related to a positive diagnosis (ASD) and vice-versa; for example low expression values of *EPHB*4 conditioned the classifier to a positive diagnosis.

## Discussion

In the present work, we applied a co-expression network-based approach to analyze gene expression data obtained from human prefrontal cortex tissue of ASD and controls (GEO experiment GSE28475). Our study combined a community detection method based on the Leiden algorithm with a machine learning approach. Using random forest and 5-fold cross-validation, we identified 41 gene communities that discriminated between controls and ASD patients with a mean accuracy higher than 85%; six of them showed an overlap between 4 and 7 genes with the SFARI database. We validated our results using an independent microarray experiment (GEO experiment GSE2852) and obtained that 38 communities reached an accuracy higher than 70%, with the best performing community having an accuracy as high as 88±3%, and with two communities, namely Comm_50 and Comm_71, that shared genes with the SFARI database and achieved an accuracy of 78±5% and 75±4%, respectively.

Our performances are consistent with those of previous studies in which gene expression signatures were identified to distinguish ASD from control subjects. Using blood microarray data (GEO experiment GSE26415) and a machine learning approach based on unsupervised methods and the support vector machine (SVM) algorithm, Oh et al found a community of 19 genes that discriminated ASD from controls with an accuracy of 93.8% [14]. Still on a blood microarray dataset (GEO experiment GSE25507), Hameed at al. performed classification by applying a combination of multiple statistical filters, wrapper methods and the SVM classifier in a 10-fold CV framework and achieved an accuracy of 86.3% [63]. On the same GEO dataset, Latkowski et al built a pipeline able to separate the autistic group from the control group exploiting both genetic approaches and machine learning algorithms. By means of a combination of SVM and RF methods they reached an accuracy of 86% and an even higher value (92.9%) on a second autism dataset (GEO experiment GSE15402) [64]. This last result outperformed the best finding of 81.8% reported by Hu et al [65].

On the two communities with potentially causal genes that we identified, we performed an eXplainable Artificial Intelligence Analysis and a preliminary biological validation.

Within Comm_50, gene EPHB4 (Ephrin Type-B Receptor 4) emerged as the most important gene for the prediction. Suppression of the expression of this gene was found to be linked to a number of disorders of cortical development, including autism and schizophrenia by Baohan et al [66]. Also mutations in gene DICER1 (Dicer 1, Ribonuclease III), the ninth most important gene in the SHAP plot for Comm_50, have been shown to increase the risk of various cancers (DICER1 syndrome) [67,68] and to be related to delays in overall development (global developmental delay), macrocephaly, ASD and various physical abnormalities [69–73]. The same gene, DICER1, was overexpressed in the dorsolateral prefrontal cortex of schizophrenia patients [74]. Within Comm_71, it is worth mentioning the pivotal role of FOXB1 (Forkhead Box B1) a gene that has been shown to be involved in neuronal development [75,76]. Moreover, about 70% (ACHE, GABRB3, PPP1R9B, PBX1) and 50% (POMT1, RFX4) of the SFARI genes contained in Comm_50 and Comm_71, respectively, were among the top twenty genes of our SHAP analysis, thus providing independent support to our analysis.

Our analysis of the over representation of KEGG pathways identified a significant pathway in Comm_50, the glycosylphosphatidylinositol-anchor biosynthesis pathway, whose dysfunction is associated with various neurological disorders, including autism spectrum disorder, multiple sclerosis and schizophrenia [77]. The same analysis in Comm_71 pinpointed a significant over representation of the fatty acid elongation pathway. There is growing evidence that disturbances in fatty acid metabolic pathways may have an impact on nervous system function and play a role in the development of autism spectrum disorders [78].

Despite the favorable results of our methodology, certain limitations must be recognized. First, the comparatively small sample sizes of the training and validation datasets may significantly limit the generalisability of our results. Although the classification performance was robust, larger and more diverse cohorts are needed in future research to further validate the predictive accuracy and stability of the identified gene communities. In addition, our study relies on transcriptomic information obtained from postmortem brain tissue. Future research could benefit from combining other data sources, including blood-derived transcriptomic profiles, to inform brain-based studies. Finally, we used SHAP to improve the interpretability of the models. Although it retains nonlinear interactions and relationships learned from tree models, standard SHAP explanations are additive, wherein higher-order interactions are not uniquely differentiated, but rather are included in the individual feature contributions. This can lead to an oversimplification of complex gene-gene interactions of the discovered communities. Given this limitation, we apply higher-order techniques in a parallel study that can reveal further interactions between the genes of the found communities.

By combining complex network analysis, machine learning, and model interpretability, our study not only achieves strong predictive performance but also contributes biologically interpretable hypotheses about ASD pathophysiology. This integrative framework, to our knowledge, is among the first of its kind applied to autism and sets a foundation for future refinement and biological exploration.

In conclusion, with our SHAP analysis we were able to detect the pivotal role of known causal variants in the obtained model, thus giving independent support to our analysis. Our preliminary biological validation also showed encouraging results consistent with the existing literature. A complete analysis of functional and pathway enrichment however is outside the scope of our research. Further analysis on the restricted number of genes in the identified communities may uncover the mechanisms responsible for autism spectrum disorder.

## Supporting information

A1 Appendix Lists of the gene communities with the best ASD-control classification performance.
(PDF)

S1 Table Classification performances of the best 41 gene communities on the training set.
(PDF)

S2 Table Classification performances of the best gene communities validated on the independent test dataset.
(PDF)
