# Supplementary material for: A joint complex network and machine learning approach for the identification of discriminative gene communities in autistic brain
Source: PLoS One. 2025 Nov 5;20(11):e0334181. doi: 10.1371/journal.pone.0334181 (PMC12588478; doi:10.1371/journal.pone.0334181)
Supplement: A1 Appendix — Lists of the gene communities with the best ASD-control classification performance. (PDF) [file pone.0334181.s001.pdf]

Lists of the 41 communities with the best ASD-control classification performance.

In the following section, we provide a list of the genes in each of the 41 gene communities with the best ASD-Control classification performance:

- **Comm_8**: SOD1, UNC50, ATP5H, GRSF1, RPL37A, ACTB, ATP1A1, TUBA6, RPS17, IMP3, C2ORF30, TXNDC14, MRPL51, GNB1, NONO, LOC401286, PPT1, SRPRB, CDKN1B, TUFM, DPYSL3, RAB10, RPS20, RHOA, CETN2, NRBP2, FEZ2, FAM82C, RAB7, PA2G4, PFDN5, ZFP36L1, CSNK2B, RPS3A, LEPROTL1, SRRM1, SEC23B, SRP9, B2M;
- **Comm_9**: CRSP2, PSG3, CAGE1, TAAR8, IL1RN, FGF23, NLRP8, C14ORF85, UGT2B4, ZNF549, GAGE1, RASA2, C15ORF43, HIST1H4G, HTR3E, FKBP6, PLAUR, RDM1, CD3D, C10ORF72, OR7G3, ADAM21, FLJ40298, TM4SF19, FAM120C, ZNF552, HIST3H3, MAP4K1, DEFB105A, C15ORF2, LOC643812, OR10A2, C1GALT1C1, ZNFN1A3, CHEK1, MYF5, C21ORF62, ANGPTL3, C1QTNF2, NFATC3, ADAM2, APOL2, PDCL2, DGAT2L6, OR4N4;
- **Comm_11**: RIBC2, FLJ25976, MYL4, MRGPRX4, OR10G3, IL17RE, TAS2R1, ZNF550, NEK3, OR4D6, ZBTB12, GPR152, NEUROD4, ABCA9, LOC652888, BAIAP2, MAFG, SART2, GABRR1, LOC389257, SLC36A2, LALBA, OR2T4, C17ORF50, HOXA13, ADRB3, ZDHHC11, KIAA1026, OR2S2, SDHAP3, CTRB2, OR4F3, RABEP2, BACH1, OR7E91P, LTA, OR11H12, SP6, PADI3, DKFZP434I1020, CYP11B1;
- **Comm_12**: ROCK2, C10ORF58, LOC729603, ORC6L, DDX51, RPL7L1, F2R, C19ORF31, CLUAP1, LOC642947, FLJ44124, SLC44A4, SLC16A12, C8ORF37, C14ORF24, C5ORF28, C9ORF80, CD52, NAG18, AIRE, CDAN1, DAPP1, SLC6A16;
- **Comm_13**: ERN1, RBP3, KRTAP13-4, CLDN17, OR2AG2, GDF2, LOC196913, ZBTB32, RLN3R1, KCNG4, DEFA5, LTB4R2, SLC13A2, TLX2, MAP1LC3A, NOS2A, C10ORF62, GPR157, SLC2A7, SIM2, UNQ3045, CCR4, CYP26C1, RUTBC2, FGFBP1, SRY, CDKN2B, TMPRSS4, KRTAP23-1, VN1R2, DUSP10, EDN2;
- **Comm_16**: AQP12A, MAP2K7, TOP1P1, PAQR5, CYLC2, KCNB2, UBE2G1, SLC16A6, ACOT2, AHNAK, SPANXC, KLC4, EDNRB, THAP3, MFAP3L, LOC92345, SEPTIN 6, MAGEA8, SLCO3A1, MYLK, C17ORF81, FTSJ3, SCRIB, C15ORF44, LOC647043, C10ORF25, PXN, HLRC1, ITGA4, ST6GALNAC2, CRB2;
- **Comm_17**: RECQL, DUSP13, NIT1, WDSUB1, PLA2G3, COQ2, HOXB6, CBX3, AK1, CTPS, XPO7, PRRG4, FUT6, RBM3, NAB2, NAPE-PLD, KRT8, TFPI2, ECAT11, EPR1, CHMP4A, LOC81691, ZNF431, TOP1P2, TPM4, CAMKK2, NHN1, LOC338328, SDC1, FLJ22624, GAGE7B, NUCKS1, TNFRSF10A;
- **Comm_19**: PIK4CA, PEBP1, C13ORF21, PRRT2, TMOD1, CCDC28A, ZFYVE20, CALM1, P15RS, CST3, KIF5C, ZNF447, LMTK3, CAMK2N1, AP3M2, BSCL2, TEX2, WASL, MT3, CXX1, SLC25A42, BLCAP, CAPZA2, CHD8, BASP1, DPYSL2, TSPAN7, RGS11, TMEM50B, ATP6V1D, IGSF4, KLHL3, UBP1, ATP6V0A1;

- **Comm_24**: FAM62B, HCRTR1, ZNF572, PURG, C15ORF40, MGC16372, AQP5, ATE1, LOC643253, GPR109B, TNFSF13, PEX12, ERBB4, C3AR1, TMEM34, FBXO25, NUF2, TNXA, TBX1, PDK1;
- **Comm_29**: ZNF80, IBSP, PAX3, CCDC50, KCNMB3, VN1R4, KRTAP19-7, 7A5, E2F7, DEFB107A, KRT20, TGIF1, PDC, WDR63, OR6V1, FLJ45910, RFXDC1, CCNJ, AMZ1, CCDC33, TTTY6, GP2, CASP7, LOC442425, MAGEB4, GATA6, HNF4A, LOC646600, DEFB124, C4ORF18, MRGPRX2, PCGF3, C1ORF186, MGC48628, EREG, THEG, ADAM20, FMO6P, ACTBL1, TMC2, OR2L3, C20ORF71, SIGLEC7, OR2T6, GATA4, NALP1, PGBD2, C10ORF119, MAGEA12, FCRL5, CFHR5, LOC400499, TRIM31, GDF9, UGT1A6, BSND, LCE2B, GPR78, RNF41;
- **Comm_30**: C1QTNF1, C17ORF64, OR2T10, CSRP2BP, DDO, C14ORF54, FAM47C, OR1G1, PCDHA6, GPR125, TCF7, ENPP7, SSX4B, PC-3, DEPDC5, C15ORF48, HOXD8, MGC44505, ENTPD8, BNC1, KRTAP1-1, DDR2, DSPG3, DDX31, LOC646182, BMP4, LOC441931, PASD1, DYRK1A, ASCL3, LOC440093, IQCF1, ZIM3, ANGPTL7, DSC1, LCE2A, LPAL2, LRRC52, GRHL3, KRTAP4-5, MUC20, TP73, HIST1H1T, SERPINA13;
- **Comm_31**: CLC, CEACAM4, TEX14, TXNDC8, LCE1F, TACR1, OR9G9, ASCL4, TDGF3, KRTAP5-5, ASB17, PLAC1, TAZ, LOC343066, SIRPB2, MEOX1, MSMB, MAD2L1BP, OR52H1, FAM47B, WTAP, SPATC1, LOC653499, MMP21, MCM10, AVPR1B, PAX4, PRH2, HIST1H3B, MSRB3, GGTLA4, HIST1H2AK, ASB4, GIMAP6, FAM69A, FAM40B, C19ORF40, MAWBP, FAM9C, HIST1H2AM, XAGE5, G6PC2, SPANX-N4, PRR5, SPINK4, PCDHA12, SLC27A6, LOC653316, MYH4, KIAA1924, FMO6;
- **Comm_33**: ITGA3, VAT1, CD63, MCL1, CD99, IGLL1, SERPINA1, ITGB7, HIG2, SPR, KIAA0586, SLC25A10, ADORA2B, FLVCR, TTK, GBP3, TSPAN14, C14ORF94, CARD10, MXD3, CTSL2, GIMAP2, DPH5, CMTM7, NNMT, PROCR, RAD54L;
- **Comm_34**: GTF2H3, RPL7A, GANAB, CBX1, PRPSAP2, MIS12, PSMD10, CD151, ASAM, NSUN5B, APH1A, ANLN, MCM4, XPO5, P8, HBP1, ZNF384, RARSL, FBXO8, CHCHD3, SMAP, U2AF1, STARD4;
- **Comm_43**: ANKRD33, C21ORF94, OR1E2, TRAT1, PRELID2, LOC346673, IFNA13, TIGD6, KIAA1920, TBC1D3C, SIGLEC11, NRK, ELOVL3, CXCL6, HYDIN, C1ORF124, ODF1, GLIS3, C13ORF26, A4GNT, MAP3K3, KCNJ11, CYP7A1;
- **Comm_45**: OR51G1, MIST, NMNAT2, HELB, CYP2C19, EDARADD, AURKAIP1, KATNAL1, CTDP1, TITF1, PTPN7, CSN1S1, LCN10, NUDT6, POU2AF1, GPR81, STAMBP, SMR3A, VWA1, PTPRC, CAND2, STXBP6, LRP3, MGC61598, PITX2, DUX3, IL29, RNPC1, OPN1LW, FLJ36779, DKFZP564N2472, OR5AY1, TTYH2, SCN1B, KRTHA5;
- **Comm_46**: ASGR2, L3MBTL2, GOLPH2, MGC50559, A1BG, ZNF595, OR10H4, LGMN, TP53BP2, PPIL3, POT1, TPM3, C14ORF173, C3ORF37, CENTA1, FLJ44894, ZNF100, TMEM102, STEAP3, INHBC, GYG2, ZNF273, MRPL44, ABCC1, LDB3, ZNF765, ARHGAP8, GPR20, LILRA6, CHST4, ZNF808, SEPN1, GNPTAB, AMICA1, COL9A3, CLEC4F, DYNLL2, DCP1B, VPS41, GTF2IRD2P, APH1B, ITLN2, PCDH17, CPZ, ZNF554, HELT, STAP2, KRTAP10-2, EPB41L1;

- **Comm_49**: WDR77, NUTF2, PKIG, DLG1, RICTOR, HLA-DRB6, PLEKHJ1, NME1-NME2, PCLKC, TDRD3, C9ORF23, PAIP2, KCTD17, POLR2K, BRD4, KIAA1274, SPSB2, ALDH16A1, L1CAM;
- **Comm_50**: MGC40222, DICER1, PSKH1, FOXE3, LSM14B, CRB3, SPERT, EPHB4, PTGES2, ACHE, PEX5L, GABRB3, PPP1R9B, SLC26A11, FLJ46536, MGC24381, PBX1, RUFY3, HMGN3, B4GALNT4, LOC283874, LOC286526, LOC653906, KLF16, OLIG3, FOXN1, PTK2B, RXRG, GANC, CACNA2D4, OSBPL1A, SNRP70, PIGW, SYF2, PVR, NR4A1, GIYD2, GPR150, ZNF706, DCTN1, VPS24, WAC, PRKCBP1, KIAA0514;
- **Comm_51**: CACYBP, SIDT2, C3ORF9, XPNPEP3, IL10, HYPK, HSPC268, TMEM156, GPR1, CHRNA5, LEP, RPL3, LOC401019, FCAR, IL21R, LOC653490, ZNF600, SHROOM4, RPL18, BOLA2, RPLP2, SLC25A5, RUSC1, MDH2, IL18, DMC1, GNB2L1, LRRFIP1, LOC649946, ARL16, PDSS2, LOC643057, KCNH6, COX4I1;
- **Comm_52**: SPIN1, ATP6AP1, DCTN4, RABEPK, ZNF289, ATP5B, OAZ1, PSMC5, PSMB1, K-ALPHA-1, RPL11, CPSF2, VPS37A, UBB, CHCHD2, TMEM107, EEF2, CD81, RPS11, PNKP, PLD1, RSRC2, IFT74, WDR18, CLSPN, FASTK, TPI1, DEDD2, MGC35295, HMGCS1, MRPL17, FIBCD1, COX5A, GCN1L1, ZHX2, C1ORF50, RPS15A;
- **Comm_53**: PCMT1, PH-4, EIF4A2, PJA2, STAT2, KIF1A, WBP2, PSAP, RDH14, EVL, SPRY2, STRC, TMED3, FLJ20558, TMEM126B, KLHDC3, HDDC2, ELLS1, RAP1GA1, SYNJ2BP, ARL6IP5, TTC10, TIMM22;
- **Comm_55**: PARK7, NCOA4, UBADC1, FSCN1, BCAP31, ACTG1, NEDD8, TMEM41B, SFRS5, CDC2L6, RPS4Y1, SEPTIN 9, CNDP2, PRR13, CDC37, XPNPEP1, CCDC56, RCD-8, MTSS1, HES6, NDUFA4, SLC12A9, ST13, C14ORF4, SSNA1, ZNF672, PPP1CA, TJAP1, ARD1A, CSPG2, CSK;
- **Comm_58**: DKFZP762E1312, CPB2, PRSS2, FLJ40629, SLC25A24, IRF4, TNPO1, GTSE1, GPX2, SLC45A2, SMC4, RAD51, MUC16, IRX5, MELK, RPS14, MAGEA1, MYO1G, C19ORF48, KIF20A, BAIAP2L1, HOXB7, TM4SF5, CEP55, FLJ13391, ESM1, LMNA, LEFTY2, APOA1, CCL20, ADM2, GDF15, APOA2, CHI3L2, TUBB6;
- **Comm_59**: HIST1H2BJ, ALPP, DKFZP564K142, MRPL27, LOC643516, EOMES, PRCC, OR2H2, MGC16703, LOC723972, PHF5A, HIST2H4A, AFF1, MGC13017, LOC134997, RPS2, SPTLC1, FLJ40411, TDP1, LOC402057, ATAD4, TAL2, HMG1L1, TWIST1, ARHGAP17, DSCR6, GK2, C1ORF112, ATP5I, LASS4, AP2S1, SLC3A2, LOC112714, LOC647760, C6ORF89, NACAP1, ZBP1, ZBTB40, TLE6, USP6, EEF1A1, ETNK2, OR3A4, MBTPS1, RPS4Y2, C20ORF191, HNRPU, PTTG2, RPL36A, SMA4, DENR, PLSCR1, FLJ32447, HIST3H2A;
- **Comm_60**: WDR58, ISLR, HOXC9, ITGB4BP, USP1, EIF5A, ZNF398, C8ORF33, PRKCZ, GRB7, ICOSLG, MTA2, ZFPM1, ZNF259, PTPLB, SCNN1B, SFMBT2, SNHG3-RCC1, HBA2, MDM1, OR11L1, CDC34, C17ORF70, SIPA1, CLDN6, GSTZ1, CENTD2, L3MBTL, GPR56, FLJ43339, TNPO2, SCGN, FHL3, LRRC8E;
- **Comm_62**: TSPO, ARL6IP4, HNRPL, SEPTIN 4, SLC45A3, GALC, MARCKSL1, GJA12, FA2H, RPL12, ENPP2, TSPAN15, RYK, BCL7B, CTNNA3, WDR45L, CD9, LOC644096, NKX2-2, PACS2, HAPLN2;

- **Comm_63**: SNAI3, OR6B1, AZI1, CHEK2, POLR1A, AMPD2, FREM2, USP43, TRIOBP, CATSPER1, SDK1, DERL3, IGSF11, CASP2, LGICZ1, MAML1, KRT18, SLC6A9, PARVB, CALML5, SLC12A6, MADCAM1, DDR1, LOXL1, ST3GAL1, CDK5RAP2, CCNDBP1, LOC649159, IL17B, DAZ2, ZNF665, LOC653762, COLEC12, CLU, EPHA10, KRT13, GNAT1, CSF3, FCER2;
- **Comm_64**: ABCC2, ASF1B, SALL4, FST, NSUN5C, CEP27, ESPL1, DNTT, LAD1, RN7SL1, RNASE4, FOLR1, C22ORF18, E2F2, PRR11, SPTA1, CDC25C, SLD5, FAM64A, SPANXA1, FLJ90166, CA9, KCNJ12, HOXB2, ALG1, APPBP1, SERPINA11, TFF1, C4ORF34, SIX6, PLA2G2D, CART1, ZNF577, HAK, HOXD13, SERTAD3, FGA, MGC2463, C16ORF60, LOC643905, MLANA, ARL8A, SGOL1, UBE2I, LOC653328, CTCFL, B3GN-T6, MYO1E, LRRN5;
- **Comm_65**: TRIM68, MGC3121, GLE1L, RPL35A, LMOD3, NDRG1, C1ORF176, C11ORF63, HIF1A, FN3KRP, ZNFX1, FZD7, WARS2, SLC38A6, LSM12, DCLRE1C, WBSCR18, GEMIN4, MKNK2, INADL, SEC11L1, ECHDC3, CREB1, SGSH, TXNIP, ET, VCP, SYVN1, EXOSC7, ZNF394, DDOST, TMSB10, C3ORF31, ZNF142, CAD, CCDC12, C3ORF34, ACTR5, ZADH1, SMARCA4, MGEA5, SEMA3E, MED19, ARMC7, UNG2, TBC1D2, MGC2654, DCP1A, LSM16, CLN3, ITPKC, DHCR7, FAM119A, CKAP4, LPXN;
- **Comm_66**: UGT2A1, MDN1, CR1, CTBP2, STK6P, FLJ35848, TRPM8, RPL35, C21ORF24, N4BP2, SPO11, C1ORF14, FABP2, PERF15, C10ORF61, HBG1, ART4, PPP2R3A, PTPLAD2, AFP, PTK6, HIST1H4D, TPH1, C21ORF66, POTE8, GNRHR, TNS4, CAPSL, C17ORF57, WDR5, SPTLC3, LOC344657, OCM, IL2RA, OR13C5, UPP1, GYS2, GATA3, IL15, GUCY2D, IL27, MMP20, RNASE10, SGPP2, SCTR, LOC401137, DEFB121, HAO1, GSG1, SILV, UGT2B10, LRRC24, PTPN22, LOC284274, UTS2, SPZ1, PRDM14, IFNA1, CASC2, OR2T35;
- **Comm_67**: INSL4, CCBE1, CTAG1B, NTF5, NPY6R, RTP2, FGG, PCDHA13, FLJ40365, KIAA1432, KRTAP12-4, LOC389827, SCGB3A1, IGFBP1, LOC257358, UNQ6411, ART5, PGAM1, LILRA3, KRT14, C12ORF25, TSPAN32, TUBA4, KRTAP4-7, CSNK1G3, SLC16A13, OR6N2, TAF11, PNLIP, LCK, BCDO2, DEFB122, DPT, OR1K1, CASC1, NEK5, UMOD, RAET1L, VSIG9, SYNGR1, TCEB3C, PKD1L2, EDG6, P2RY10;
- **Comm_69**: SNAI1, CCNT2, PRM3, PRKACG, ZNF238, FSD1CL, PADI1, SLA, SIT1, FLJ16331, KRT2A, FRG2, METTL6, TSPY1, GTF2A1, C10ORF120, PABPC3, RCN1, SPACA4, LOC594834, HOXC10, FZD5, KRTAP3-1, SLC26A5, SEMA6B, PSMAL, PF4, PPIL6, FBXL5, OVOL1, LCE1A, CHRNE, LOC653216, VPREB3, EN1, TANK, BPY2, DNAJB14, DAZ1, CYP17A1, KRTAP5-7, C21ORF99;
- **Comm_70**: CENTG3, BLZF1, C11ORF38, RNUXA, GSDML, ADD1, SLC25A37, POLR2C, CDIPT, FKBP8, TCP1, HIVEP2, CAPZB, CCDC104, SGCB, PQBP1, PSCD1, ATPBD3, DOK4, DAPK3, NUDT22, IGSF8, SORL1, ADCK1, ALKBH6, SAMD10, ADORA3, PANK1, GABARAP, LOC375449, MAFF, LYPD1, CD59, VHL;
- **Comm_71**: PMFBP1, ENPP3, KRT6E, APOH, C14ORF153, CFC1, FAT2, MGC52498, GALE, PVRL4, MGC39681, DEFB123, LOC124220, ACTRT1, LOC441242, TNP1, ZP2, FLJ40504, C1ORF118, INSL5, DKFZP547H025, POMT1, RFX4, TTLL10, EHHADH, LOC343521, FAHD1, XKRX, SDR-O, FOXB1, MYOCD, FRK, PGR, VPS13C, IAPP, SLC10A6, FTMT, ARS2, PRAMEF16, ADAMTSL1, MMD2, CNGB1, KIAA0853;

- **Comm_73**: RPS10, FAM27L, RAB23, ERMAP, KRTAP12-1, AMN, CRYGB, LOC653703, ARID5A, CHX10, LOC653285, KCNG2, MT1B, IFIT1L, C20ORF134, OR10Q1, KRTHA8, HIST2H3C, ZNF32, SPACA5B, MYADM, FLJ25369, PRSSL1, CYS1, SIRPG, FCHSD1, FLJ14816, SPANX-N1, LPIN3, MGC39581, RBMS1, UBE2NL, TREML2, CD200R1, C17ORF83, ELA2A;
- **Comm_78**: NBR2, LOC399900, DNAI2, KIF4A, HBD, AKR1D1, ZNF430, SHCBP1, FOXM1, HOXD3, POP1, FAM10A4, OR52A5, C17ORF74, OR52N2, TMPRSS3, NKX2-3, LOC647881, MAGEB3, LOC285453, FLJ41423, TOMM7, CDK5RAP3, ASB10, MGAM, CORO7, CCDC27, ACTL8, MME, GPR62, ANAPC11, CRYAA, MOGAT2, IL15RA, SPIB, GP1BA, HCG9, PHLDB3, KRT7, FERD3L, WNT2B, LOC146909, PSMC1, SLC6A19, EIF3S6, LOC91561, SLC7A3, LOC401296, VMAC, IRX1, C20ORF10, C18ORF24, BAZ2A, LOC441251, EVX2, LDLRAD3, LUC7L, LRRC18;
- **Comm_80**: CRKL, RPL27A, MKLN1, EPRS, RPS25, KIAA1191, COX5B, PUM1, PCNP, RPL38, FTHL7, UCRC, RPL30, REV1, CYORF15A, AKR1B1, MRPS22, SORT1, ARIH2, CHCHD1, RBM12B, RPL19, RSBN1, TACC1, COQ5, PTDSS1, JARID1B, KIAA1147;
- **Comm_83**: FLJ46688, KNTC2, IMPG2, C1ORF105, FOXA1, RNF128, AKAP3, RHAG, ME3, LOC554251, NBPF10, CCRL1, TRAF3IP2, BRCA2, CTAGE5, IRS4, CLEC3A, CTAG2, OR7C2, OR4B1, C6ORF10, CRTAM, SLC6A2, GLRX2, IFNA8, MS4A13, OR2A2, GPR128, HIST1H3G, IQGAP3, WDR72, MAGEB6, LOC653466, GLYAT, RNASE12, SPACA3, FLJ20581, C6ORF78, TAAR1, OR8H3, FANCD2, C4BPB, CHRNG, LHX4, CDCA2, HRG, HMGA2, NBPF14, MAT1A, KLRD1, SPANX-N3, TPTE2, XG, TBX22, OR4S1, OR13C4, PPP1R8, NFYA, MAS1L, LOC128153, OR6C2, SPRR2D;
- **Comm_84**: SGK2, SMAD1, ROBO1, OR5B21, PTGIR, DLX4, PCDHB11, HOXB13, LRRC43, ADAMTSL5, S100A3, CD79B, CD300E, OR1N2, KIAA1171, C1QL4, LOC91353, FLJ32926, TIMM50, GPR32, VEGF, COL18A1, IGFL1, SYT15, BCL11B, PLA2G2F, HIST1H2BO, ATP11A, PLAA, PSEN1, CD300C, PEBP4, DPPA2, FOXN4, PRDM15, OTP, SOCS7, APOL3, EBAG9, PRB4, CHRNA2, VPS16, LRRC46, FASN, GPBAR1, MKI67, CYP19A1, DACT1, RAB37, EPHA2, NPAS3, AKR1A1, ZWINT, MPN2, OR1C1, MC3R, CBFB, PDZK1IP1;
- **Comm_85**: SNX5, C19ORF24, C20ORF117, LOC651076, CASP3, BICD2, RPS6KA2, P4HA2, SCARF1, DNAJC3, KCNC4, ZNF580, IFI6, C2ORF16, PRMT1, ALDOA, ABL1, DPPA4, WNT9B, APBB1, ADAM33, CCDC9, ADAR, RFP2, PSD3, ACOT11, GRIK4, SREBF1, PITPNM3, SFT2D2, PHF2, TMEM1, GJB1, ASMTL, RASSF2, HNRPA3, PDIA6, PDDC1, TRAFD1, SEMA3F, FXR1.
