# Supplementary material for: A joint complex network and machine learning approach for the identification of discriminative gene communities in autistic brain
Source: PLoS One. 2025 Nov 5;20(11):e0334181. doi: 10.1371/journal.pone.0334181 (PMC12588478; doi:10.1371/journal.pone.0334181)
Supplement: S1 Table — Classification performances of the best 41 gene communities on the training set. (PDF) [file pone.0334181.s002.pdf]

S1 Table. Classification performances of the best 41 gene communities on the training set. The results shown include the mean AUC, the F1 score, and the average number of genes selected by Boruta feature selection in 100 repetitions of 5-fold cross-validation. All 41 reported communities (out of a total of 86 gene communities) achieved an average classification accuracy greater than 85%, with a maximum value of $(98 \pm 1)\%$ reached by the Comm 59. In green we highlighted gene communities that shared genes with the *SFARI Gene* database.

Community	Cardinality	Accuracy	AUC	F1 Score
Comm_8	39	91.06 ± 2.05	88.81 ± 2.22	93.54 ± 1.51
Comm_9	45	97.62 ± 1.00	97.82 ± 1.39	98.24 ± 0.72
Comm_11	41	88.56 ± 2.50	86.48 ± 3.01	91.66 ± 1.83
Comm_12	23	90.12 ± 1.95	89.13 ± 2.41	92.69 ± 1.44
Comm_13	32	85.44 ± 2.17	82.64 ± 2.38	89.43 ± 1.66
Comm_16	31	90.35 ± 2.44	89.31 ± 2.59	92.86 ± 1.87
Comm_17	33	91.98 ± 1.84	92.36 ± 2.14	93.95 ± 1.39
Comm_19	34	91.48 ± 1.99	89.20 ± 2.39	93.86 ± 1.43
Comm_24	20	85.87 ± 2.93	83.01 ± 3.63	89.77 ± 2.12
Comm_29	59	93.37 ± 1.42	93.11 ± 1.69	95.07 ± 1.06
Comm_30	44	90.79 ± 1.49	88.03 ± 1.87	93.41 ± 1.07
Comm_31	51	93.33 ± 1.45	92.70 ± 1.75	95.08 ± 1.07
Comm_33	27	93.27 ± 1.66	93.12 ± 2.45	95.00 ± 1.19
Comm_34	23	88.44 ± 1.80	87.11 ± 2.38	91.47 ± 1.30
Comm_43	23	92.60 ± 1.35	91.41 ± 1.98	94.59 ± 0.95
Comm_45	35	88.10 ± 2.18	86.87 ± 2.85	91.19 ± 1.57
Comm_46	49	90.12 ± 2.28	88.88 ± 2.58	92.72 ± 1.70
Comm_49	19	87.37 ± 1.87	85.70 ± 2.39	90.70 ± 1.36
Comm_50	44	94.46 ± 1.25	92.47 ± 1.43	96.02 ± 0.91
Comm_51	34	95.21 ± 1.88	93.31 ± 2.25	96.56 ± 1.36
Comm_52	37	95.67 ± 1.62	94.33 ± 1.89	96.87 ± 1.17
Comm_53	23	86.94 ± 1.95	84.16 ± 2.25	90.56 ± 1.44
Comm_55	31	87.92 ± 2.33	86.94 ± 2.76	91.01 ± 1.74
Comm_58	35	89.33 ± 2.21	88.65 ± 2.77	92.05 ± 1.62
Comm_59	54	98.10 ± 1.19	97.99 ± 1.36	98.60 ± 0.88
Comm_60	34	90.63 ± 1.86	89.10 ± 2.09	93.15 ± 1.38
Comm_62	21	85.04 ± 2.21	83.56 ± 2.16	88.86 ± 1.77
Comm_63	39	87.56 ± 2.03	86.33 ± 2.01	90.76 ± 1.57
Comm_64	49	91.06 ± 1.84	88.46 ± 2.42	93.59 ± 1.30
Comm_65	55	95.65 ± 1.67	94.30 ± 2.17	96.86 ± 1.21
Comm_66	60	96.73 ± 0.82	96.31 ± 1.18	97.60 ± 0.60
Comm_67	44	91.12 ± 1.99	89.39 ± 2.31	93.53 ± 1.46
Comm_69	42	89.19 ± 1.94	88.71 ± 1.99	91.91 ± 1.50
Comm_70	34	89.77 ± 1.81	87.85 ± 2.25	92.55 ± 1.31
Comm_71	43	93.81 ± 1.44	92.79 ± 1.49	95.46 ± 1.09
Comm_73	36	91.13 ± 2.35	89.47 ± 3.30	93.55 ± 1.65
Comm_78	58	93.25 ± 1.70	90.87 ± 2.12	95.17 ± 1.21
Comm_80	28	85.63 ± 2.44	82.88 ± 2.73	89.57 ± 1.80
Comm_83	62	94.52 ± 1.59	94.33 ± 2.18	95.94 ± 1.15
Comm_84	58	86.02 ± 2.01	84.33 ± 2.39	89.67 ± 1.50
Comm_85	41	85.94 ± 2.17	83.98 ± 2.57	89.66 ± 1.62
