# Supplementary material for: A joint complex network and machine learning approach for the identification of discriminative gene communities in autistic brain
Source: PLoS One. 2025 Nov 5;20(11):e0334181. doi: 10.1371/journal.pone.0334181 (PMC12588478; doi:10.1371/journal.pone.0334181)
Supplement: S2 Table — Classification performances of the best gene communities validated on the independent test dataset. (PDF) [file pone.0334181.s003.pdf]

S2 Table. Classification performances of the best gene communities validated on the independent test dataset. The table reports the mean classification accuracy, the AUC and the F1 score for the independent data set. The results were obtained by averaging more than 100 repetitions of the 5-fold cross-validation procedure, with estimated errors indicated. Here are reported the 38 (out of the previous 41) communities achieved an accuracy higher than 70%, with a maximum value of $(88 \pm 3)\%$ reached by the Comm 78. In green we highlighted gene communities that shared genes with the *SFARI Gene* database.

**Community**	**Accuracy**	**AUC**	**F1 Score**
Comm_3	75.78 ± 4.59	75.78 ± 4.59	77.10 ± 4.94
Comm_4	77.28 ± 3.49	77.28 ± 3.49	78.24 ± 3.24
Comm_6	72.28 ± 4.33	72.28 ± 4.33	72.95 ± 4.29
Comm_7	70.78 ± 3.47	70.78 ± 3.47	71.00 ± 3.86
Comm_12	75.22 ± 4.30	75.22 ± 4.30	76.00 ± 4.37
Comm_16	78.72 ± 5.37	78.72 ± 5.37	79.91 ± 5.49
Comm_20	70.39 ± 3.58	70.39 ± 3.58	71.47 ± 3.69
Comm_21	70.61 ± 3.89	70.61 ± 3.89	71.79 ± 3.84
Comm_25	84.44 ± 4.27	84.44 ± 4.27	84.69 ± 4.11
Comm_34	71.56 ± 3.62	71.56 ± 3.62	73.08 ± 3.95
Comm_35	71.56 ± 6.31	71.56 ± 6.31	73.53 ± 6.71
Comm_36	73.44 ± 4.42	73.44 ± 4.42	75.09 ± 5.22
Comm_39	82.33 ± 3.40	82.33 ± 3.40	82.97 ± 3.44
Comm_40	70.33 ± 5.40	70.33 ± 5.40	72.34 ± 5.28
Comm_41	78.61 ± 4.17	78.61 ± 4.17	78.93 ± 4.19
Comm_43	79.33 ± 4.01	79.33 ± 4.01	80.54 ± 3.75
Comm_44	81.22 ± 3.87	81.22 ± 3.87	81.59 ± 3.75
Comm_46	73.83 ± 2.81	73.83 ± 2.81	74.83 ± 2.46
Comm_47	71.78 ± 3.57	71.78 ± 3.57	72.28 ± 3.82
Comm_48	73.44 ± 3.47	73.44 ± 3.47	74.30 ± 3.54
Comm_50	78.33 ± 4.86	78.33 ± 4.86	78.76 ± 5.18
Comm_51	74.50 ± 4.19	74.50 ± 4.19	75.02 ± 4.93
Comm_52	76.50 ± 4.93	76.50 ± 4.93	78.12 ± 5.06
Comm_53	73.44 ± 4.24	73.44 ± 4.24	75.38 ± 4.23
Comm_57	74.94 ± 3.95	74.94 ± 3.95	76.46 ± 4.21
Comm_59	77.61 ± 4.64	77.61 ± 4.64	79.16 ± 5.04
Comm_65	74.33 ± 4.61	74.33 ± 4.61	74.85 ± 4.26
Comm_70	70.50 ± 4.38	70.50 ± 4.38	72.04 ± 4.79
Comm_71	75.44 ± 4.40	75.44 ± 4.40	76.74 ± 4.94
Comm_72	73.67 ± 4.50	73.67 ± 4.50	74.47 ± 4.40
Comm_75	78.83 ± 3.50	78.83 ± 3.50	79.61 ± 3.66
Comm_76	73.11 ± 3.87	73.11 ± 3.87	74.25 ± 4.21
Comm_77	73.89 ± 4.42	73.89 ± 4.42	75.21 ± 4.52
Comm_78	88.33 ± 3.55	88.33 ± 3.55	89.16 ± 3.30
Comm_79	76.33 ± 4.60	76.33 ± 4.60	77.15 ± 4.68
Comm_80	77.28 ± 3.75	77.28 ± 3.75	78.00 ± 4.20
Comm_82	78.83 ± 3.84	78.83 ± 3.84	79.88 ± 3.50
Comm_86	75.78 ± 4.20	75.78 ± 4.20	77.02 ± 3.93
